# Diurnal Variation in Outcomes of Percutaneous Coronary Intervention

**DOI:** 10.7759/cureus.7677

**Published:** 2020-04-15

**Authors:** Jahanzeb Malik, Nismat Javed

**Affiliations:** 1 Cardiology, Rawalpindi Institute of Cardiology, Rawalpindi, PAK; 2 Internal Medicine, Shifa College of Medicine, Shifa Tameer-E-Millat University, Islamabad, PAK; 3 Internal Medicine, Weill Cornell Medicine, New York, USA

**Keywords:** primary percutaneous intervention, primary angioplasty, myocardial infarction, off hours

## Abstract

Objectives

To determine whether the outcomes differ during regular hours as compared to off hours in patients with acute myocardial infarction who undergo primary percutaneous coronary intervention.

Methods

We conducted a prospective, interventional study of patients who presented to a specialized cardiac care center. Patients who satisfied the inclusion and exclusion criteria were included in the study. They were divided into two groups so that one group received intervention during regular working hours while the other group received intervention during 'off' hours. The data was collected through a self-constructed questionnaire. Cronbach's alpha was used to assess the internal consistency of the questionnaire, and it was found to be 0.75. The data obtained was analyzed on IBM's Statistical Package for the Social Sciences (SPSS) version 21.0 (IBM, Armonk, NY).

Results

Out of 100 participants, 64 (64%) were males and 36 (36%) were females. The mean age of the participants was 58.58 ± 13.21 years. Most (44%) of the patients were diabetic. Inferior wall myocardial infarcts were more common in our study. Percutaneous intervention during 'off' hours was associated with more adverse outcomes. The differences in gender and angina requiring revascularization were statistically significant (p-value<0.05).

Conclusion

No significant difference in outcomes was observed in both groups of patients. Metacentric data from different institutes should be gathered for a comprehensive insight on this topic where door-to-balloon times and initiation of catheterization lab procedures are practiced in different ways.

## Introduction

Acute myocardial infarction is an important cause of morbidity and mortality in the developing world [1]. Percutaneous coronary intervention is a common cardiovascular procedure [2]. Primary percutaneous coronary intervention, a mechanical intervention that enables restoration of blood flow by reopening the occluded artery via a catheter with an inflatable balloon, is currently the preferred reperfusion option for acute myocardial infarction with ST-segment elevation [3].

Acute ST-segment elevation myocardial infarction is a major manifestation of coronary artery disease. It is associated with a high incidence [4]. Primary percutaneous coronary intervention is better than thrombolysis for acute ST-segment elevation myocardial infarction and a delay in treatment affects outcome. This factor is a major concern during later times of the day because facilities have to be activated and staff has to be brought in from home to ensure proper care. Some healthcare centers are, therefore, concerned that favorable outcomes may be difficult to achieve for patients presenting after normal working hours [5].

The outcome of percutaneous coronary intervention may be influenced by several factors, for example, the annual number of procedures, the experience of the operating physician, time delays in treatment and organization level of myocardial infarction care [6]. These factors do not seem to play a role in overall long-term mortality [7].

Several studies have been conducted but limited data is available regarding this topic in Pakistan. The objective of the study is to assess the impact of diurnal variation in the outcome of primary percutaneous coronary intervention among patients visiting a specialized cardiac care center in Rawalpindi, Pakistan.

## Materials and methods

It was a prospective, interventional study conducted at a specialized care center in Rawalpindi. The duration of the study was six months and the sample size was 100 patients. This sample size was calculated using the World Health Organization's calculator. The sampling technique used was simple random sampling. Patients who were willing to participate in the study were included in the study. Patients who presented with acute myocardial infarction that had evolved after 12 hours were excluded from the study. 

The participants were randomly divided into two groups with 50 patients each. This randomization process was done using a software. Patients in Group A were treated during the first half of the day (8:00 am to 8:00 pm) while patients in Group B were treated during the second half of the day (8:00 pm to 8:00 am).

After induction, the patients were referred to the procedure as soon as the procedure room was available. Primary percutaneous coronary intervention was performed as recommended by guidelines. All patients received intravenous heparin. Pre-dilation, administration of glycoprotein IIb/IIIa inhibitors, thrombus aspiration and stent implantation were performed at the discretion of the surgeon. The use of intra-aortic balloon was restricted to patients with cardiogenic shock. The participants were followed during hospitalization. Further evaluation was done on scheduled follow-up visits after discharge.

Data was collected on a self-designed questionnaire and analyzed using Statistical Package for the Social Sciences (SPSS) version 21.0 (IBM, Armonk, NY). Cronbach's alpha was used to assess the internal consistency of the questionnaire, and it was found to be 0.75. Means and standard deviations were calculated for variables such as age and ejection fraction. Frequencies and percentages were calculated for qualitative variables such as gender and efficacy. A chi-square test was used to compare the outcomes and a p-value≤0.05 was considered as significant.

## Results

There were 100 participants in the study. The mean age of the participants was 58.58 ± 13.21 years. There were 64 (64%) male and 36 (36%) female participants in the study. The demographic data according to each group is given in Table 1.

**Table 1:** Demographic data of both groups SD=standard deviation, N=frequency

	Group A (total number of participants was 50)	Group B (total number of participants was 50)
Age in years (mean ± SD)	56.94 ± 15.49	60.22 ± 10.36
Gender (N, %)		
Males	34 (68)	30 (60)
Females	16 (32)	20 (40)
Co-morbid conditions (N, %)		
Hypertension	15 (30)	20 (40)
Diabetes mellitus (type 2)	24 (48)	20 (40)
Chronic kidney disease	7 (14)	9 (18)
Cerebrovascular disease	4 (8)	1 (2)

The mean percentage of ejection fraction was 39.02 ± 7.58 for Group A and 36.76 ± 9.23 for Group B. This difference in ejection fraction was not statistically significant. The types of myocardial infarctions are shown in Table 2.

**Table 2:** Myocardial infarctions in both groups

Types of infarcts	Group A (total number of participants was 50)	Group B (total number of participants was 50)
	Frequency (%)	Frequency (%)
Anterior wall myocardial infarct	14 (28)	11 (22)
Inferior wall myocardial infarct	35 (70)	37 (74)
Lateral wall myocardial infarct	1 (2)	2 (4)

The outcomes of both groups were also evaluated. This is shown in Table 3.

**Table 3:** Outcomes of both groups

Outcomes	Group A (total number of participants was 50)	Group B (total number of participants was 50)
	Frequency (%)	Frequency (%)
Angina requiring revascularization	5 (10)	11 (22)
Re-infarction	4 (8)	4 (8)
Acute stent thrombosis	3 (6)	4 (8)
Major bleeding	4 (8)	6 (12)
Minor bleeding	1 (2)	2 (4)
Deaths	3 (6)	5 (10)

There was no statistically significant difference between the two groups in terms of outcomes. Male participants had more probability of having angina that required revascularization. This difference in probability was significant (p-value<0.05).

## Discussion

Our study aimed to assess the outcomes of percutaneous coronary intervention in patients who had presented during normal working hours (8.00 am to 8.00 pm) and during ‘off’ hours (8.00 pm to 8.00 am). The outcomes were better for the patients who had the procedure done during normal working hours (8.00 am to 8.00 pm), but the differences were not found to be significant.

The mean age of the participants was 58.58 ± 13.21 years. The usual age associated with a good outcome in percutaneous coronary intervention is 65 years [8]. In our case, the age of the participants was lesser and despite the age difference, the incidence of angina requiring revascularization (16%) was still higher. This incidence was higher for the group (11%) with intervention done during ‘off’ hours. This is further explained by the difference in the ejection fraction between the two groups.

It was found that patients who presented during the day had higher ejection fractions as compared to patients who had presented during later times of the day. This difference was not significant. Ejection fraction (less than 50%) is considered an important prognostic factor for patients with acute coronary syndromes without heart failure [9]. Revascularization techniques yield better results in patients with reduced ejection fraction [10]. The prognostic value of this factor, in the case of successive revascularization attempts, is questionable.

Inferior wall infarcts were more common in our study. Inferior wall myocardial infarction is associated with right ventricular dysfunction and the manifestation of this infarct as ST-segment elevation is still debatable [11,12]. This factor did not have a significant impact on the outcomes of the study.

The adverse outcomes were more frequently seen during the ‘night shifts’. This can be attributed to several factors. Delays could occur in diagnosis owing to limited staff as suggested by a study regarding outcomes of percutaneous intervention. In our case, the medical staff is limited to on-call doctors and nurses. This leads to a delay in diagnosis by emergency staff [13,14].

A peculiar finding of the study was that there was a significant difference between gender and angina requiring revascularization (p-value<0.05). Males (14%) were most likely to develop this complication. In our study, males were more likely to be hypertensive and diabetic. This has been established by studies that relate hypertension to a high degree of mortality in patients who had revascularization done [15,16].

There were some limitations to our study. This study had a smaller sample size and was a single-center study. The study did not determine the importance of experience of the team during 'off' hours. There were technical difficulties in measuring the door-to-balloon time. There should be multi-center studies to explore this topic further. It would also be helpful to include door-to-balloon times, time to intervention and further information on follow-up.

## Conclusions

The time of patient presentation and treatment has been shown to be associated with differences in clinical outcomes for various medical procedures. This observation may reflect variability in patient characteristics and pathophysiology and in the availability of experienced hospital staff. The latter consideration may influence both the quality and expediency of treatment. Metacentric and randomized control trials should be done throughout hospitals to accurately describe any difference in outcomes so that proper measures can be devised.

## References

[1] Mendis S, Thygesen K, Kuulasmaa K (2011). World Health Organization definition of myocardial infarction: 2008-09 revision. Int J Epidemiol.

[2] Amin AP, Crimmins-Reda P, Miller S (2018). Novel patient-centered approach to facilitate same-day discharge in patients undergoing elective percutaneous coronary intervention. J Am Heart Assoc.

[3] Ozaki Y, Katagiri Y, Onuma Y (2018). CVIT expert consensus document on primary percutaneous coronary intervention (PCI) for acute myocardial infarction (AMI) in 2018. Cardiovasc Interv Ther.

[4] Mumma BE, Kontos MC, Peng SA, Diercks DB (2014). Association between prehospital electrocardiogram use and patient home distance from the percutaneous coronary intervention center on total reperfusion time in ST-segment-elevation myocardial infarction patients: a retrospective analysis from the National Cardiovascular Data Registry. Am Heart J.

[5] Sorita A, Ahmed A, Starr SR (2014). Off-hour presentation and outcomes in patients with acute myocardial infarction: systematic review and meta-analysis. BMJ.

[6] Dhungel S, Malla R, Adhikari C (2018). Door-to-balloon time and the determining factors in a tertiary cardiac center in Nepal. Indian Heart J.

[7] Dharma S, Dakota I, Sukmawan R, Andriantoro H, Siswanto BB, Rao SV (2018). Two-year mortality of primary angioplasty for acute myocardial infarction during regular working hours versus off-hours. Cardiovasc Revasc Med.

[8] Deng J, Wang X, Shi Y, Zhao X, Han Y (2018). Prognostic value of the age, creatinine, and ejection fraction score for non-infarct-related chronic total occlusion revascularization after primary percutaneous intervention in acute ST-elevation myocardial infarction patients: a retrospective study. J Interv Cardiol.

[9] Agra RB, Cordero A, García-Acuña JM (2018). Determinants and prognostic impact of heart failure and left ventricular ejection fraction in acute coronary syndrome settings [Article in English, Spanish]. Rev Esp Cardiol (Engl Ed).

[10] Abouzaki NA, Exaire JE, Guzmán LA (2018). Role of percutaneous chronic total occlusion interventions in patients with ischemic cardiomyopathy and reduced left ventricular ejection fraction. Curr Cardiol Rep.

[11] Albulushi A, Giannopoulos A, Kafkas N, Dragasis S, Pavlides G, Chatzizisis YS (2018). Acute right ventricular myocardial infarction. Expert Rev Cardiovasc Ther.

[12] Bischof JE, Worrall CI, Smith SW (2018). In inferior myocardial infarction, neither ST elevation in lead V1 nor ST depression in lead I are reliable findings for the diagnosis of right ventricular infarction. J Electrocardiol.

[13] Isogai T, Yasunaga H, Matsui H, Tanaka H, Ueda T, Horiguchi H, Fushimi K (2015). Effect of weekend admission for acute myocardial infarction on in-hospital mortality: a retrospective cohort study. Int J Cardiol.

[14] Tang L, Chen PF, Hu XQ, Shen XQ, Zhao YS, Fang ZF, Zhou SH (2017). Effect of Chinese national holidays and weekends versus weekday admission on clinical outcomes in patients with STEMI undergoing primary PCI. J Geriatr Cardiol.

[15] Saluveer O, Redfors B, Angerås O (2017). Hypertension is associated with increased mortality in patients with ischaemic heart disease after revascularization with percutaneous coronary intervention - a report from SCAAR. Blood Press.

[16] Fokkema ML, James SK, Albertsson P (2016). Outcome after percutaneous coronary intervention for different indications: long-term results from the Swedish Coronary Angiography and Angioplasty Registry (SCAAR). EuroIntervention.

